# Case report: Removal of a subcutaneous implantable cardiac defibrillator in a pediatric patient with hypertrophic cardiomyopathy after a septal myectomy. Insights on current indications of type of ICD in children with hypertrophic cardiomyopathy and left ventricular tract obstruction

**DOI:** 10.3389/fped.2022.932390

**Published:** 2022-09-14

**Authors:** Paola Dolader, Iosune Alegria, Patricia Martínez Olorón, Joaquin Fernandez-Doblas, Ferran Gran, Ferran Roses-Noguer

**Affiliations:** ^1^Department of Paediatric Cardiology, Vall d'Hebron University Hospital, Barcelona, Spain; ^2^Department of Paediatric Cardiology, Complejo Hospitalario de Navarra, Pamplona, Spain; ^3^Department of Paediatric Cardiac Surgery, Vall d'Hebron University Hospital, Barcelona, Spain; ^4^Department of Paediatric Cardiology, Royal Brompton and Harefield NHS Foundation Trust, London, United Kingdom

**Keywords:** hypertrophic cardiomyopathy, sudden cardiac death, subcutaneous implantable cardiac defibrillator, transvenous implantable cardioverter defibrillator, myectomy

## Abstract

Hypertrophic cardiomyopathy is a heart muscle disease with an annual incidence between 0.24 and 0.47/100000 in childhood. Sudden cardiac death is the most common cause of death in this population. Although some medical treatment can decrease the risk of sudden cardiac death, implantable cardioverter defibrillator continues to be the most reliable treatment. Different types of devices and programming strategies can be used in patients with hypertrophic cardiomyopathy depending on each center and specific patient condition. We report a pediatric patient affected with hypertrophic cardiomyopathy who had and ICD implantation in primary prevention. Four years later he developed symptomatic left ventricular outflow tract obstruction and a surgical septal myectomy was performed. After the myectomy the patient developed complete left bundle branch block on his 12 lead ECG, and unfortunately none of the S-ICD vectors were suitable after the myectomy and it had to be explanted and replaced for a new transvenous ICD.

## Introduction

Sudden cardiac death (SCD) is the most common cause of death in pediatric patients with hypertrophic cardiomyopathy (1). Implantable cardioverter defibrillators (ICD) are indicated in patients with worse phenotype presentation to reduce the risk of sudden cardiac death. However, these devices are not exempt of complications such as infection, lead failure or inappropriate shocks (2) and the decision to implant an ICD on primary prevention warrants a careful consideration. In addition, different type of ICD technology is available including transvenous ICD, subcutaneous-ICD, or hybrid epicardial ICD, all of them have their advantages and disadvantages. Deciding which technology is best for our patients is usually very complex and it is based on the patient characteristics and the center/operator preference (3).

We report a pediatric patient diagnosed with hypertrophic cardiomyopathy with a subcutaneous ICD implanted on primary prevention who developed a severe symptomatic left ventricular outflow tract obstruction a underwent a surgical septal myectomy. After the surgery, his ECG showed a complete left bundle branch block, not present before the myectomy. The S-ICD vector analysis after the myectomy showed that none of the 3 possible vectors was suitable anymore due to low R:T ratio and/or high R-wave. These factors could not be modified with programming and unfortunately the S-ICD had to be explanted and a new transvenous ICD implanted (Table 1).

**Table 1:** Timeline.

**Time**	**Important clinical data**
2014	Diagnosis of hypertrophic cardiomyopathy
July 2016	Subcutaneous ICD implantation
October 2016	Inappropriate shock
December 2018	Myectomy
December 2018	Subcutaneous IC explanted, transvenous ICD implanted

This case report highlights the importance of considering avoiding the implant of a S-ICD in children with hypertrophic cardiomyopathy with severe left ventricular outflow tract obstruction that might require a future septal surgical myectomy causing inevitably the presence of left bundle branch that can lead to S-ICD not being suitable.

## Case description

A fourteen-year-old boy with the diagnosis of hypertrophic cardiomyopathy was referred to our hospital for surgical septal myectomy due to severe symptomatic left ventricular outflow tract obstruction.

Our patient was diagnosed at the age of ten years old after his paternal uncle had a sudden cardiac death while he was doing exercise. He had always been asymptomatic before the event. Uncle's necropsy revealed left ventricular hypertrophy in keeping with the diagnosis of hypertrophic cardiomyopathy. His brother, our patient's father, underwent an ECG and an echocardiography which was consistent with hypertrophic cardiomyopathy. He also had genetic testing which showed a missense pathogenic variant in the MYH7 gene (p. Arg663Cys). Following these results, all his sons were transferred to the outpatient cardiology clinic for evaluation. Three of his sons, including our patient, tested positive for the familial mutation, being affected the three of them. Our patient eldest brother had an ICD implanted for primary prevention.

Our patient clinical results included an ECG that showed normal sinus rhythm with no conduction abnormalities, high voltages, marked Q waves in lead III and abnormal repolarisation pattern with flattened T waves in left precordial leads. His echocardiography showed a concentric left ventricular hypertrophy, with a small left ventricular cavity size with no systolic anterior movement of the mitral valve and no left ventricular outflow tract obstruction. There was no concomitant right ventricular hypertrophy. He was completely asymptomatic. He underwent a 24 h ECG which revealed no arrythmias and an exercise test which was normal.

However, over his follow-up, his echocardiogram showed a rapid progression of his hypertrophy, and he developed increased gradient across the left ventricular outflow tract. Additionally, he became symptomatic having exercise intolerance and he was started on oral propranolol 40 mg every 8 h (1 mg/kg/dose TDS), to reduce his symptoms. He had a cardiac MRI which showed presence of late gadolinium enhancement in his LV compatible with extense LV fibrosis. Giving his family history, the rapid progression of his left ventricular hypertrophy, the left ventricular outflow tract obstruction, and the LV fibrosis on MRI, he underwent subcutaneous ICD implantation on primary prevention 2 years after the diagnosis. Before the ICD implant, he had a full ECG vector analysis at rest and on exercise with 2 out of 3 vectors suitable for an S-ICD implant. The implant was performed using a 2 incisions technique and S-ICD lead was implanted on the left parasternal region. S-ICD programming after implant showed 2 out of 3 vectors had excellent R:T ratios and no T wave oversensing was seen at rest and exercise. Device programming included one conditioning zone at 210 bpm and a VF zone at 230 bpm. The patient was discharged home 24 h later.

During his follow up, 3 months later he had an inappropriate shock for oversensing, and the ICD vector was changed from alternate to secondary.

**Figure 1:**
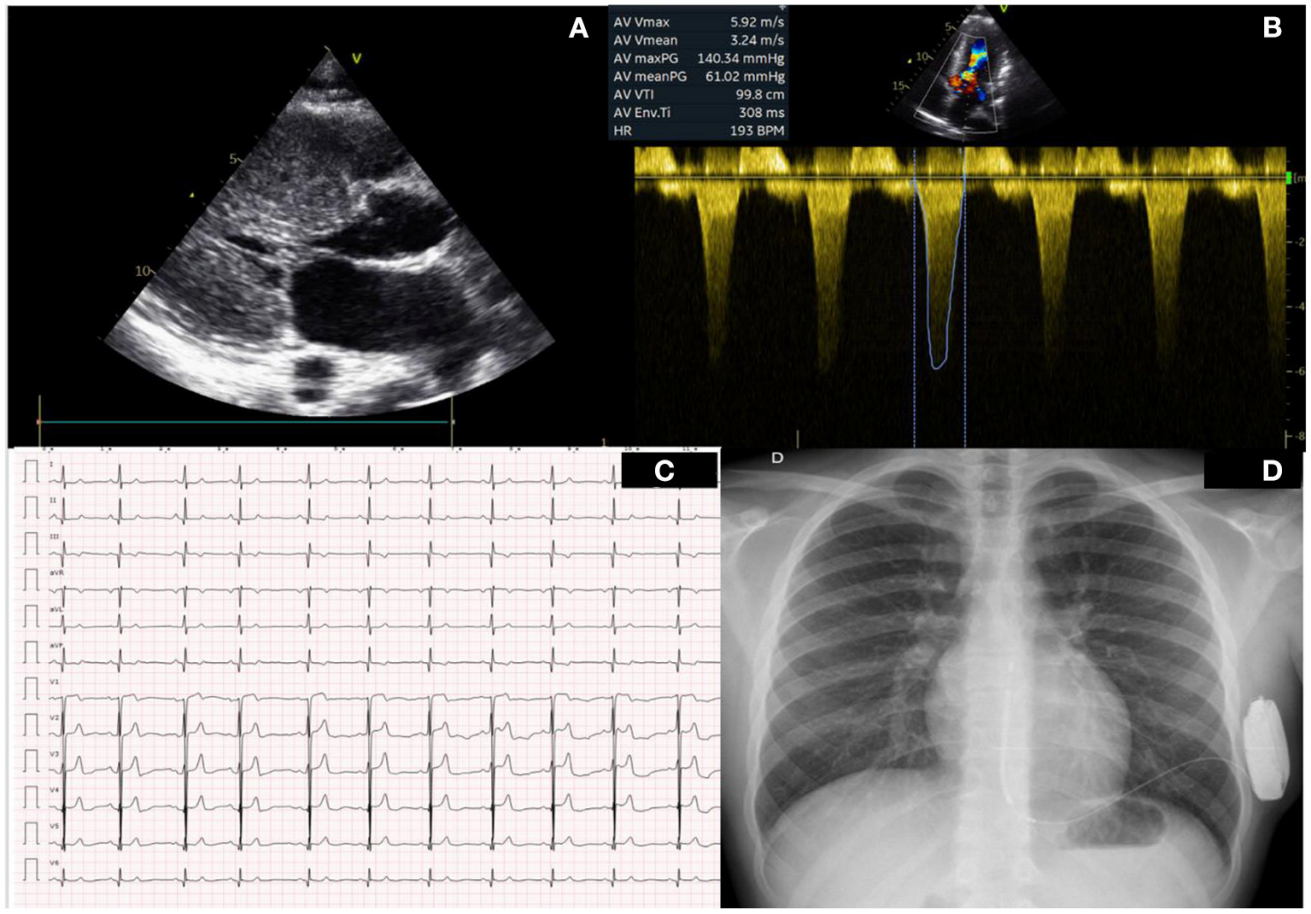
Baseline clincal test pre surgery. **(A)** Left parasternal long axes showing severe left ventricular hypertrophy. **(B)** Doppler across the left ventricular outflow tract showing a peak gradient of 140 mmHg. **(C)** Baseline ECG before surgery. **(D)** Chest X Ray with SICD in place.

**Figure 2:**
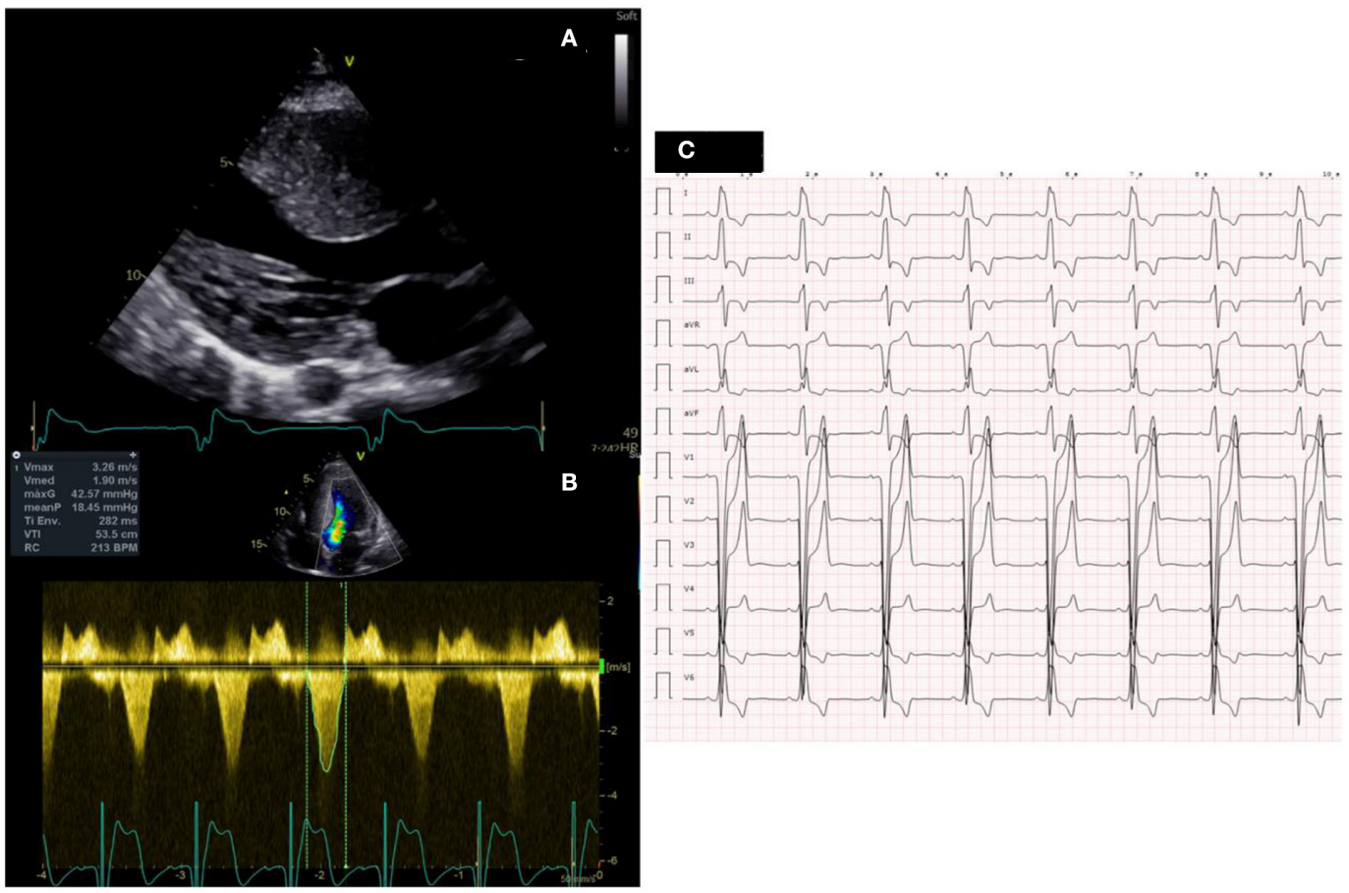
Clincal test after surgery. **(A)** Left parasternal long axes showing significant reduction of interventricular septal thickness. **(B)** Doppler across the left ventricular outflow tract showing a peak gradient of 42 mmHg. **(C)** ECG after surgery showing the presence of a complete left bundle brunch block.

**Figure 3:**
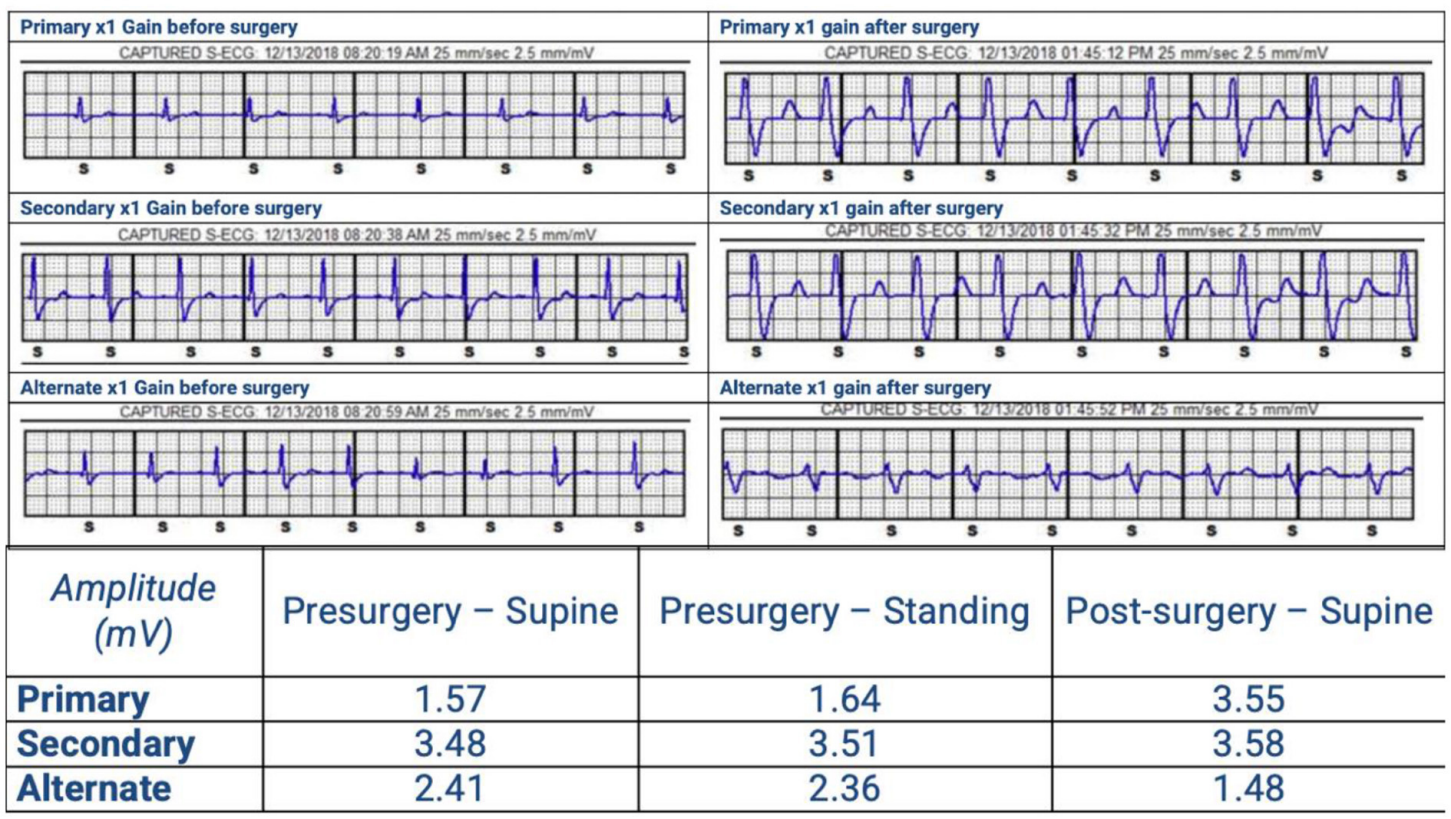
SICD vector analysis pre and after surgery. The primary vector failed due to high R-wave (out of range) and the secondary and alternate vectors failed due to low R:T ratio.

When he was fourteen years old, he started to feel dizzy while doing low intensity exercise. There were no arrhythmias documented with his symptoms. His echocardiograms showed an increasing of his left ventricular outflow gradients. For that reason, he was then transferred to our hospital for considering surgery to relief his left ventricular outflow tract obstruction. His ECG was as previously described. His echocardiogram showed a maximal wall thickness of 35 mm (Z Score + 12) at the level of the interventricular septum and a posterior wall of 20 mm (Z Score + 6.3). He had a complete systolic anterior movement of the mitral valve with moderate mitral regurgitation. The left atrium was mildly dilated (Area 23.5 cm2 Z score + 2.3). There was flow acceleration across the left ventricular outflow tract with a peak gradient over 140 mmHg and mean gradient of 60 mmHg at rest (see Figure 1). The patient was discussed in our Surgical MDT and a decision was made to perform a surgical septal myectomy to improve his symptoms. A resection of the septal hypertrophy was performed uneventfully, and the lead of the subcutaneous ICD was then replaced in the exact same position as it was before the surgery. There was a significant reduction of the left ventricular outflow gradients. However, his ECG changed completely after surgery presenting a complete left bundle branch block as seen in Figure 2. The day after the surgery, S-ICD interrogation showed S-ICD vectors sensing was no longer valid. The S-ICD was not able to complete automatic setup because there was a change in the electrogram morphology. The primary vector failed due to high R-wave (out of range) and the secondary and alternate vectors failed due to low R:T ratio (Figure 3). At that time, the only option to avoid the risk of inappropriate shocks was to explant de S-ICD and implant a new ICD system. Before the surgery, we carefully considered all the different ICD options. A new S-ICD implant was discarded due to the risk of inappropriate shocks. Implanting an epicardial ICD was considered an option (4), but it was rejected because it would imply a second sternotomy for our patient, also because of the limited life duration of epicardial leads and finally because it would imply not being able to do further MRIs. In the end, the patient underwent an explant of his old S-ICD and an implant of a new dual chamber, single coil transvenous MRI compatible ICD system.

After the surgery, the patient improved considerably his symptoms. During the 3 years follow up period, the patient had been clinically well with excellent exercise capacity, his ECG showed complete left bundle branch, and his echo demonstrated only a moderate peak gradient of 40 mmHg through his LVOT. The transvenous ICD had no appropriate or inappropriate shocks.

## Discussion

Hypertrophic cardiomyopathy has an annual incidence between 0.24 and 0.47/100,000 in pediatric population (1). Complications such as heart failure and SCD are well-described, being sudden cardiac death the most common cause of death in children affected with hypertrophic cardiomyopathy (1–2 vs. 0.8%/year) (1). In fact, children with hypertrophic cardiomyopathy seem to be at higher risk of developing malignant arrhythmias (5). ICD has proved to be a safe and effective therapy preventing SCD in these patients. However, this therapy is not exempt of complications and risk stratification is vital to assess the need for an ICD in primary prevention (6). Our patient underwent an ICD implant when he was 12 years old, 2 years after his initial diagnosis, based on a primary prevention Class IIA indication. It was decided to offer an S-ICD to reduce the risks of transvenous leads and because at the time his LVOT was only moderate. PACES recent published guidelines recommend ICD implant with a Class I indication to all pediatric patients with HCM who are survivors of sudden cardiac arrest or have sustained ventricular tachycardia. Class IIA indication is recommended for children with HCM who have >1 primary risk factors, including unexplained syncope, massive left ventricular hypertrophy, non-sustained VT, or family history of early HCM-related SCD (7). Additionally, there are some unique considerations in pediatric patients, including the duration of the device and leads, the size of the patient relative to the device, the particularities of the phenotype of each patient, the increased physical activity, and the different psychological impact of ICD related complications. Therefore, the potential life-saving performance of ICD devices must be weighted individually against all these concerns. Regarding risk stratification, a pediatric SCD risk model has been published recently by Norrish et al. in which unexplained syncope, non-sustained ventricular tachycardia, maximal wall thickness and left atrium diameter were associated with SCD (8).

Once is decided a patient can benefit from ICD therapy, device choice and programming strategies are important in determining long-term outcome, especially in young patients. A wide variety of approaches, including transvenous ICD, epicardial ICD and S-ICD, are seen depending on each center and patient needs (3). Nowadays, more simple devices are preferred, if possible, to decrease the risk of important complications. Long-term complications mainly include system infection, lead malfunction or displacement and the delivery of inappropriate shocks (2). All these concerns are accentuated in a young patient cohort facing decades of future risk. Is for that reason that subcutaneous ICD represents an important evolution in ICD therapy by positioning the lead in the subcutaneous layer of the thoracic cage, thereby avoiding potential complications related to the wear of transvenous leads (9). Subcutaneous ICD has the advantage of less serious complications regarding infection and lead failure. However, it does not provide bradycardia pacing or anti-tachycardia pacing and its risk of T wave oversensing is still a challenge. A second option is to consider a transvenous ICD, which can provide anti-tachycardia and bradycardia treatment, which can be indicated in some patients and can reduce the LVOT obstruction with RV apex pacing. Nevertheless, the risk of endocarditis, lead displacement or lead fracture is higher comparing with subcutaneous ICD. Furthermore, risks of removing leads with a transvenous ICD are higher than with a subcutaneous ICD (10–12). An Epicardial ICD system is also an option in children with HCM. It allows pacing, anti-tachycardia treatment and good DFT thresholds even in teenagers. Unfortunately, they require a thoracotomy, or a sternotomy and the leads lifetime duration is less compared to TV leads. Still, it must be considered as the first option when the patients are <15 kg, or a cardiac operation is planned.

Another important complication is the risk of inappropriate shocks. The incidence of inappropriate shocks does not depend on the type of device (13). T-wave oversensing is the main cause for inappropriate shocks with a subcutaneous ICD, as described in our patient. To qualify for S-ICD implant, patients should undergo a thorough screening procedure to ensure that their QRS and T-wave morphology and amplitude are analyzed in various positions. If the T-wave is too large or delayed in relation to the QRS complex, then there is a risk of T-wave oversensing and double counting of the heart rate, which can cause inappropriate therapy. Therefore, baseline ECG screening on the right and left parasternal position at rest and during exercise is crucial to ensure SICD is a safe option. However, baseline ECG in a patient with hypertrophic cardiomyopathy may change, due to the progressive nature of hypertrophy. Both the QRS and T-wave may change in amplitude and morphology as the ventricle hypertrophies, and this must be kept in mind. Moreover, in those patients with severe left ventricular outflow tract obstruction that might require surgery to relief left ventricular outflow tract obstruction, their ECG will necessarily change dramatically causing difficulties in vector sensing with the S-ICD that could not be overcome with programming and thus, leading to SICD explant.

**Figure 4:**
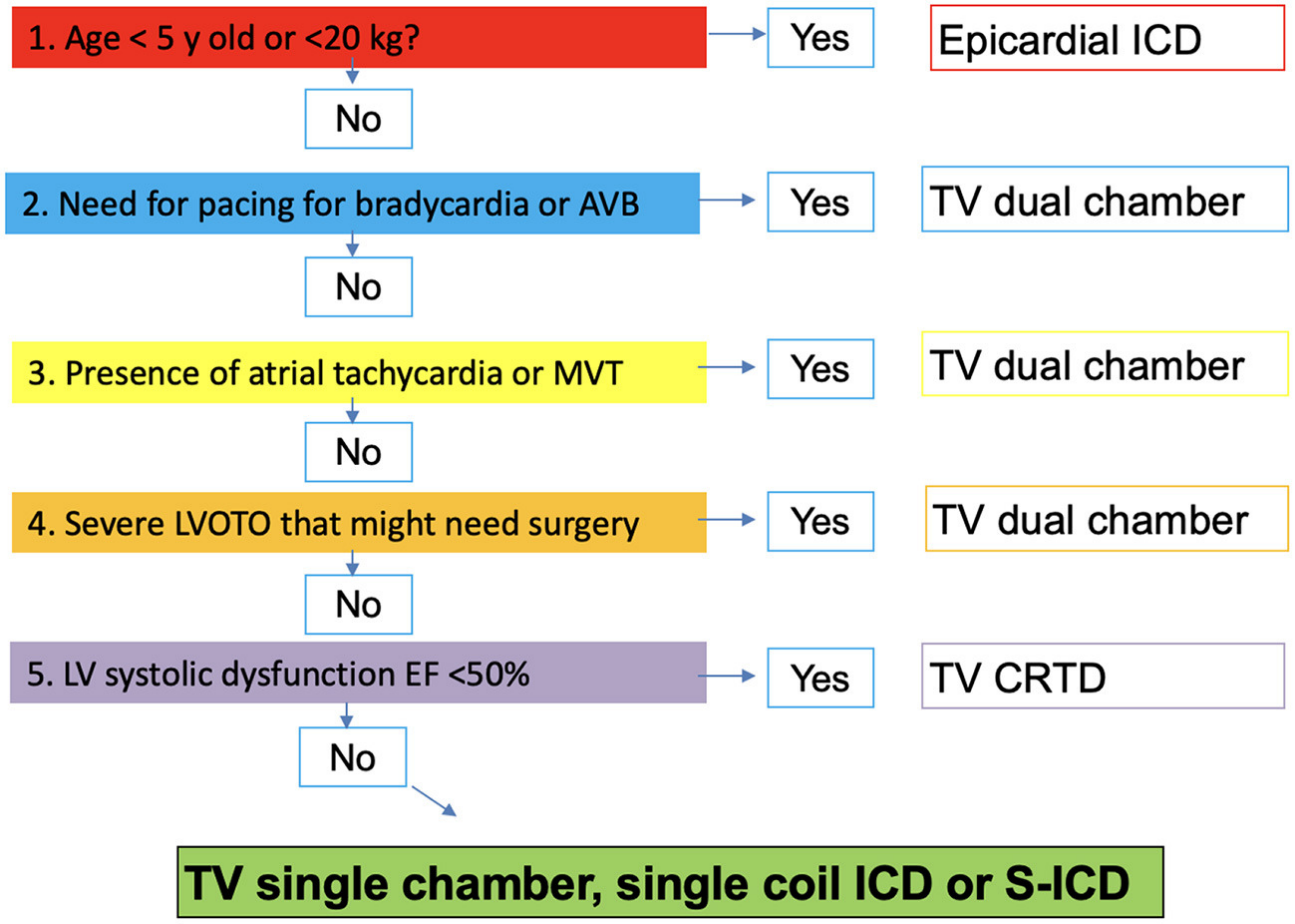
Stepwise approach to guide selection of type of ICD implant in children with hypertrophic cardiomyopathy. ICD, implantable cardiac defibrillation; AVB, Atrioventricular block; TV, Transvenous; MVT, Monomorphic Ventricular tachycardia; LVOTO, Left ventricle outflow tract obstruction; CRTD, Cardiac resynchronisation therapy with defibrillation; S-ICD, Subcutaneous ICD.

In the Figure 4 we propose a stepwise approach to guide the decision-making process on which type of ICD should be implanted in a child with HCM depending on age and their clinical phenotype.

In conclusion, ICD therapy has significantly decreased the incidence of sudden cardiac death in patients with hypertrophic cardiomyopathy. Different programming strategies and devices can be used depending on each center and specific patient situation. S-ICD has been proposed as a good option with less serious complications. However, baseline screening ECG is crucial to avoid T-wave oversensing that can lead to inappropriate shocks. Surgery to relief left ventricular outflow tract obstruction is needed in some patients to improve symptoms, and it can be more frequent in young patients, as the progression of hypertrophy can developed during adolescence. After the surgery, complete left bundle branch block on baseline ICD is not infrequent. This fact increases the risk of S-ICD malfunction and could cause inappropriate shocks due to T-wave oversensing. In our case these changes were impossible to overcome with programming and the S-ICD had to be explanted and replaced for a conventional transvenous device. This case report highlights the importance of considering avoiding subcutaneous ICD in those patients with high gradients across the left ventricular outflow tract in which myectomy can be needed.

## Data availability statement

The raw data supporting the conclusions of this article will be made available by the authors, without undue reservation.

## Ethics statement

Ethical review and approval were not required for the study on human participants in accordance with the local legislation and institutional requirements. Written informed consent to participate in this study was provided by the patient legal guardian for the publication of any potentially identifiable images or data included in this article.

## Author contributions

Manuscript revision: FR-N, FG, JF-D, and PM. Manuscript preparation: PD, FG, and FR-N. Case report design: PD, IA, and FR-N. All authors contributed to the article and approved the submitted version.

## Conflict of interest

The authors declare that the research was conducted in the absence of any commercial or financial relationships that could be construed as a potential conflict of interest.

## Publisher's note

All claims expressed in this article are solely those of the authors and do not necessarily represent those of their affiliated organizations, or those of the publisher, the editors and the reviewers. Any product that may be evaluated in this article, or claim that may be made by its manufacturer, is not guaranteed or endorsed by the publisher.
